# Surgical treatment of malignant involvement of the inferior vena cava

**DOI:** 10.1186/1477-7800-3-19

**Published:** 2006-08-16

**Authors:** Patrizio Castelli, Roberto Caronno, Gabriele Piffaretti, Matteo Tozzi, Chiara Lomazzi, Gianlorenzo Dionigi, Luigi Boni, Renzo Dionigi

**Affiliations:** 1 General Surgery-Department of Surgical Sciences, Insubria University Hospital, Versa, Italy

## Abstract

**Background:**

Resection and replacement of the inferior vena cava to remove malignant disease is a formidable procedure. The purpose of this review is to describe our experience with regard to patient selection, operative technique, and early and late outcome.

**Methods:**

The authors retrospectively reviewed a 12-year series of 11 patients; there were 10 males, with a mean age 57 ± 13 years (range 27–72) who underwent caval thrombectomy and/or resection for primary (n = 9) or recurrent (n = 2) vena cava tumours. Tumour location and type, clinical presentation, the segment of vena cava treated, graft patency, and tumour recurrence and survival data were collected. Late follow-up data were available for all patients. Graft patency was determined before hospital discharge and in follow-up by CT scan or ultrasonography. More than 80% of patients had symptoms from their caval involvement. The most common pathologic diagnosis was renal cell carcinoma (n = 6), and hepatocarcinoma (n = 2). In all but 2 patients, inferior vena cava surgical treatment was associated with multivisceral resection, including extended nephrectomy (n = 5), resection of neoplastic mass (n = 3), major hepatic resection (n = 2), and adrenal gland resection (n = 1). Prosthetic repair was performed in 5 patients (45%).

**Results:**

There were no early deaths. Major complications occurred in 1 patient (9%). Mean length of stay was 16 days. Late graft thrombosis or infection did not occur. The mean follow-up was 22.7 months (range 6–60). There have been no other late graft-related complications. All late deaths were caused by the progression of malignant disease and the actuarial survival rate was 100% at 1 year. Mean survival was 31 months (median 15).

**Conclusion:**

Aggressive surgical management may offer the only chance for cure or palliation for patients with primary or secondary caval tumours. Our experience confirms that vena cava surgery for tumours may be performed safely with low graft-related morbidity and good patency in carefully selected patients.

## Background

Neoplastic caval involvement has traditionally been suspected by the presenting symptoms of lower extremity swelling and venous engorgement [1]. The widespread use of preoperative CT scans has demonstrated that many asymptomatic patients could have involvement of the vena cava by tumour [2].

However, resection and replacement for malignant involvement of the inferior vena cava (IVC) is generally indicative of advanced disease, and has been performed rarely because of the magnitude and risk of the operation; nevertheless, in the absence of surgical resection, patient survival is limited, because effective treatment alternatives are few [2].

Experience with this type of operation at an individual institution remains very limited [2-4]; indeed, most publications on the subject have been sporadic single-case reports. In our experience, a variety of tumours were encountered; partial resection of the caval wall, with either primary or patch closure, has been reported to be preferable to graft replacement because it is safer and easier to perform [2].

The aim of this retrospective analysis is to describe the outcome of surgical treatment for IVC malignant involvement in a series of 11 patients with extensive abdominal tumours and to discuss our policy for IVC replacement.

## Materials and methods

In the last 15-years, 11 resections of the IVC were carried out to achieve excision of malignant or benign abdominal tumours. Procedures involving lateral IVC resection, transcaval removal of tumour thrombus, with or without cardiopulmonary bypass (CPB) were included. The patient's clinical presentation, the type and location of the tumour, the segment of vena cava replaced, graft patency, and tumour recurrence and survival were collected for all patients [Tab. 1]. There were 10 men with a mean age of 57 ± 13 years (range, 27–72; median 60).

**Table 1:** Demographic data of 11 patients with IVC neoplastic involvement: Insubria experience

*sex/age*	*symptoms*	*type of tumour*	*IVC segment*	*months: follow-up*
M/66	lower limb oedema	kidney	infrarenal	60: dead
M/55	lower limb oedema	kidney	infrarenal	60: alive
M/58	lower limb oedema	kidney	infrarenal	12: dead
M/71	pneumonia-pulmonary embolism	kidney	infrarenal	12: dead
M/60	lower limb oedema	kidney	infrarenal	24: dead
M/64	lower limb oedema	hepatocarcinoma	right heart involvement	12: dead
F/49	acute heart failure	pelvic leiomyomatosis	right heart involvement	12: alive
M/72	weight loss	kidney	infrarenal	6: alive
M/27	fatigue/weight loss	testicular teratocarcinoma	infrarenal	12: dead
M/60	pain/weight loss	hepatocarcinoma	suprarenal	12: alive
M/45	lower limb oedema	adrenal gland	suprarenal	6: alive

The indication for IVC resection was primary invasion by adjacent primary (n = 8) or recurrent (n = 2) malignancy. In the remaining patient, the indication was benign tumour involvement. One patient received preoperative adjuvant therapy in the form of radiation therapy.

**Figure 1:**
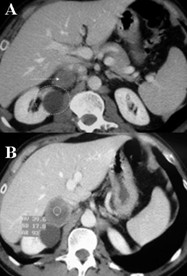
Preoperative CT-angiography shows a complete caval obstruction (arrow) due to a tumoral mass of the right adrenal gland (A); postoperative histology revealed a primary carcinoma with kidney infiltration (B).

**Figure 2:**
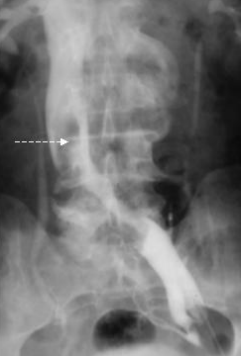
Preliminary cavography: caval involvement (arrow) from a retroperitoneal mass (pelvic leiomyomatosis).

Chest roentgenography, computed tomography (CT), ultrasonography, or magnetic resonance imaging (MRI) were used to exclude the presence of metastatic disease, to assess local resectability of the tumour, and to assess the extent of involvement and obstruction of the IVC [Fig. 1]. Early in our experience, vena cavography was used preoperatively to determine the degree of caval obstruction, and to help plan the operation [Fig. 2]. At present, vena cavography is used only if the venous anatomy is not shown well by the other imaging studies. Involvement of the IVC was classified as infrarenal (n = 7) or suprarenal (n = 2); two patients (18%) had right heart involvement. Obstruction was partial in 8 cases (73%) and complete in 3 patients (27%).

**Figure 3:**
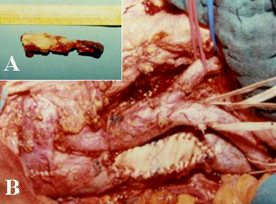
Postoperative findings: tumoral thrombus after complete surgical removal (A). Dacron patch angioplasty was used to repair IVC resection to achieve complete tumour excision (B)

If the tumour was found locally resectable, an extensive medical evaluation of the patient was performed to assess operative risk. Surgery was performed with extracorporeal circulation in two cases because of right atrial involvement. Abdominal surgical access was carried out through a subcostal incision (n = 8) or through a midline incision (n = 3). Complete gross tumour resection was achieved in all patient following the main oncological principles (no touch techniques and vessels legation at their origins). Tumor thrombectomy alone was performed in 7 patients (64%), 3 had reconstruction of the infrarenal IVC with patch angioplasty (27%), and 1 patients (9%) had graft replacement of the suprarenal IVC. Dacron grafts (Bard^®^-Tempe, AZ-USA) were used in all patients with patch reconstruction [Fig. 3], while PTFE (W.L. Gore and ass.^®^-Flagstaff, AZ-USA) was preferred for replacement. Long-term prophylactic antibiotics were administered in all patients; postoperatively, all patients started receiving subcutaneous heparin or low-dose intravenous heparin within 24 to 48 hours.

Tumour histology included renal cell carcinoma (n = 6), hepatocarcinoma (n = 2), testicular teratocarcinoma (n = 1), pelvic leiomyomatosis (n = 1), and adrenal gland carinoma (n = 1). Graft patency was systematically assessed in the early postoperative period and during follow-up by one or more studies including vena cavography, CT scan or ultrasonography. These same imaging studies were used to determine the presence of recurrent or metastatic disease. Adjuvant radiation or chemotherapy was given to selected patients depending on the tumour type.

## Results

More than 80% of the patients were initially seen with one or more symptoms from their tumour, including lower extremity oedema (n = 6; 55%), pain-weight loss and fatigue (n = 4; 36%), and pneumonia following pulmonary embolism (n = 1; 9%). In all patients, IVC resection was associated with visceral resection, including extended nephrectomy (n = 5), resection of retroperitoneal mass (n = 3), major hepatic resection (n = 2), and excision of the adrenal gland (n = 1). Negative tumour margins were obtained in all patients. Median intraoperative transfusion volume was 2 units of packed red blood cells (range 0–5 units). There were no perioperative deaths. One patient had major complication: cardio-respiratory failure developed in a 71-year male patient with myasthenia gravis, and chronic obstructive pulmonary disease with preoperative poor respiratory function. The remaining patients (91%) recovered and were discharged uneventfully. The median hospital stay was 14 days (mean 16; range, 6–32). There was no evidence of graft-related infection during the hospital stay or since discharge; CT scans or duplex studies obtained before discharge demonstrated patency of the IVC graft in all patients.

**Figure 4:**
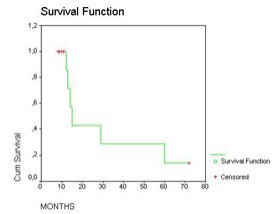
Kaplan-Meier analyses of the survival in 11 patients.

Follow-up ranged 6 to 60 months (mean, 22.7; median 12). There were no re-operations. Late deaths occurred from regional or distant metastatic disease. Despite the variety of malignancies, overall survival was calculated by using Kaplan-Meier survival estimates with 95% confidence intervals [Fig. 4]. One-year survival was 100%; mean survival was 31 months (median 15, range 6–60).

## Discussion

The role of IVC resection and replacement for the treatment of malignant disease is limited to a small number of select patients, and only few surgeons have focused on its development [5,6]. In fact, data regarding the survival of patients with primary or secondary caval malignancies is sparse [6-9]. This series describing 11 patients who underwent IVC resection for neoplasms with or without prosthetic replacement, is one of the largest to date.

In contrast to many previously published series, our experience includes patients with a variety of tumoral conditions at different levels of the IVC [2-4]. Although there is a trend toward improvement in survival in these patients, and aggressive management could produce long-term survival, the diverse aetiology of cancers herein and the small number of patients preclude conclusions regarding the impact of these operations on survival. In addition, replacement after IVC resection is controversial: however in our experience, patients with extensive intraluminal involvement, open thrombectomy alone or patch reconstruction of the IVC did not carried the risk of late recurrence from the venous wall. If radical tumour resection and caval replacement are to have any role, the operative mortality and morbidity must be low, patients must be carefully selected, and the grafts must be durable. The operative mortality in our patients is low, given the magnitude of the procedure, and is consistent with other reports, with an estimated mean survival of 31 months for patients with malignant disease. In comparison, median survival without resection has been reported to be one month for patients with primary retroperitoneal tumours [2]. Thus, we advocate more widespread use of IVC resection; indeed, we believe that a multidisciplinary approach, and careful evaluation and treatment of these patients is a mandatory component for patient selection.

Procedures have been usually performed through an abdominal incision; indeed, in agreement with previous authors, we think that cardiopulmonary bypass (CPB) is unnecessary unless the tumour extends significantly into the atrium as occurred in two cases [10,11]. Surgical procedures could be performed with single or double-staged procedures. CPB has been used to remove tumours and thrombus in the IVC and right heart cavities with success; nowadays, the single-stage caval and cardiac resection of the tumour has been abandoned due to the high risk of diaphragmatic lacerations [11]. In the presented cases, we adopted a two-stage approach because the initial thoracic allowed safe resection of the intracardiac tumour mass: moreover operative time was shorter, despite the risks of a second general anaesthesia, and could reduce the risk of bleeding because of systemic heparinization required for CPB. The slow growth of the tumour allows for a safe interval between two major surgical procedures.

Caval involvement has traditionally been suspected by the presenting symptoms of venous engorgement [1,4]. The widespread use of preoperative CT scans has demonstrated that many asymptomatic patients could have involvement of the vena cava by tumour [2,3]. Preoperative radiological investigation is critical for careful patient selection and outcome: most patients are imaged with CT scan to define the extent of the tumour and the presence of metastatic disease. In our early experience, vena cavography was routinely performed preoperatively; however, we no longer routinely use this study but reserve it for patients with suspected IVC occlusion or those with signs or symptoms of venous insufficiency for whom the IVC is not adequately imaged by CT scan or MRI. Considering symptoms and signs, wide retroperitoneal resection including a segment of the IVC disrupts pre-existing venous channels and thus can reduce collateral venous return. Our experience subverted previous evidence: indeed, early or late postoperative symptoms of venous obstruction were never observed, whereas preoperative lower limb swelling disappeared after surgery with thrombectomy and resection.

The presence of tumour thrombus into inferior vena cava from abdominal cancers carries the threat of pulmonary tumour embolus [12]. When this occurs, the outcome is catastrophic; thus, some reports have advocated placement of a suprarenal IVC filter before resection. However, reports of tumour emboli during surgical resection for malignant caval involvement are anecdotal [12]; therefore we did not place caval filter in any of our patients, and pulmonary embolism was never encountered in our experience.

## Conclusion

We believe that aggressive surgical management may offer the only chance for cure or palliation of symptoms in select patients with primary or secondary tumours of the IVC: patients with localized disease, no significant medical problems, and a good preoperative performance status should be considered candidates for tumour resection.

To date, our clinical experience has been satisfactory. The ability to safely accomplish caval resection without perioperative death and the excellent functional results in terms of patient activity are noted in our series; this trend likely reflects the strict criteria for patient selection.
